# Sexual Propagation Enhances Tea Quality Through Rhizosphere Microbiome Assembly and Metabolic Reprogramming in *Camellia sinensis*

**DOI:** 10.3390/microorganisms14091949

**Published:** 2026-09-03

**Authors:** Yu-Xiang Zhang, Li-Xian Wang, Jian-Gen Zhang, Chen Li, Luo-Fa Wu, Xin-Feng Jiang, Ye Chun

**Affiliations:** 1 Jiangxi Provincial Key Laboratory of Plantation and High Valued Utilization of Specialty Fruit Tree and Tea, Cash Crops Research Institute of Jiangxi Province, Nanchang 330045, China; a2249533424@163.com (Y.-X.Z.); wanglixian0214@126.com (L.-X.W.); zjg7087@sina.com (J.-G.Z.); hanwuji1110@126.com (C.L.); 2 Ministry of Education Key Laboratory of Crop Physiology, Ecology and Genetic Breeding, Jiangxi Agricultural University, Nanchang 330045, China; 3 Institute of Agricultural Engineering, Jiangxi Academy of Agricultural Sciences, Nanchang 330200, China; lychunzi@126.com

**Keywords:** *Camellia sinensis*, propagation method, tea quality, metabolomics, microbiome

## Abstract

Tea quality is largely determined by the accumulation of specialized metabolites in fresh leaves, yet the effects of the propagation method on tea quality and its belowground ecological basis remain insufficiently understood. In this study, sexually propagated (SR) and asexually propagated (AR) tea plants were compared by integrating soil physicochemical analysis, leaf quality and physiological measurements, widely targeted metabolomics, and rhizosphere metagenomic profiling. Compared with AR, SR plants exhibited more favorable rhizosphere nutrient conditions, with soil organic matter, total nitrogen, alkali-hydrolyzable nitrogen, and available phosphorus increasing by 23.1%, 18.2%, 27.8%, and 161.5%, respectively, although available potassium decreased by 24.4%. SR leaves also contained higher dry matter, tea polyphenol, and soluble sugar contents, which increased by 11.5%, 58.8%, and 8.6%, respectively. In addition, superoxide dismutase, peroxidase, and indole-3-acetic acid oxidase activities were 30.4%, 92.0%, and 21.7% higher under SR, whereas hydrogen peroxide content remained unchanged. Metabolomic profiling revealed marked differences in leaf metabolic composition between the two propagation types, with differentially accumulated metabolites mainly enriched in flavonoid biosynthesis, phenolic acid metabolism, caffeine metabolism, carotenoid biosynthesis, plant hormone signaling, and α-linolenic acid metabolism. Rhizosphere metagenomic analysis further showed that SR was characterized by higher relative abundances of Actinomycetota, Pseudomonadota, Planctomycetota, Alphaproteobacteria, and Streptomycetales, together with distinct microbial functional profiles related to glycolysis, the tricarboxylic acid cycle, and pyruvate metabolism. Significant correlations were identified between several SR-enriched microbial taxa and quality-related metabolites, particularly flavonoids and phenolic acids. Overall, under the present field conditions, sexual propagation was more favorable than asexual propagation for tea quality formation, as reflected by improved nitrogen and phosphorus availability, greater accumulation of quality-related metabolites, higher antioxidant enzyme activities, and distinct rhizosphere microbial carbon-metabolic potential. These findings provide an integrated soil–microbiome–metabolome perspective for understanding propagation-related differences in tea quality.

## 1. Introduction

The tea plant (*Camellia sinensis* L.) is an economically important perennial crop, and its fresh leaves provide the raw material for one of the world’s most widely consumed beverages. Tea quality is largely determined by the composition and accumulation of specialized metabolites in fresh leaves, particularly tea polyphenols, flavonoids, phenolic acids, amino acids, soluble sugars, and alkaloids [1,2]. These metabolites collectively contribute to tea flavor, aroma, nutritional value, and health-promoting properties [3]. Understanding the factors regulating the accumulation of these metabolites is therefore essential for improving tea quality [4].

Tea quality formation is influenced by a combination of genetic, environmental, and management factors [5]. Among these, propagation strategy is an important factor influencing plant establishment, development, and long-term field performance [6]. In tea cultivation systems, both sexual propagation and asexual propagation are widely employed. Sexual propagation involves seed formation through fertilization, resulting in genetically diverse offspring with potential variation in root development, physiological traits, and environmental adaptation. In contrast, asexual propagation in tea is mainly achieved through vegetative methods such as stem cuttings, in which adventitious roots develop from detached shoots and genetically identical clones are produced from selected mother plants. Asexual propagation through cuttings is generally preferred because it preserves elite parental traits and ensures population uniformity [7]. In contrast, sexually propagated plants generally possess greater genetic heterogeneity and may develop root systems and adaptive characteristics that differ from those of clonally propagated plants [8,9]. Previous studies have mainly focused on the effects of propagation methods on seedling establishment, growth performance, and stress resistance [10]. However, whether propagation strategy is associated with coordinated differences in soil properties, rhizosphere microbial communities, leaf metabolism, and ultimately tea quality remains poorly understood.

Recent advances in plant–soil ecology have demonstrated that crop quality is not solely regulated by plant genetic characteristics but is also strongly influenced by belowground ecological processes [11,12]. The rhizosphere microbiome constitutes a key biological interface linking soil environments with plant physiological and metabolic activities [13]. Rhizosphere microorganisms participate in organic matter decomposition, nutrient mineralization, phosphorus solubilization, nitrogen transformation, and phytohormone production, thereby regulating plant growth and metabolism [14,15]. Increasing evidence suggests that microbial communities have been associated with the biosynthesis and accumulation of plant secondary metabolites through nutrient mobilization and plant–microbe signaling [16]. In tea ecosystems, rhizosphere microbial composition has been reported to be closely associated with nutrient availability, tea plant health, and quality-related metabolic traits [17,18]. Furthermore, plant genotype and cultivation practices can significantly alter microbial community assembly and functional composition, indicating that plant-associated factors may be linked to tea quality through changes in rhizosphere microbial community composition and function [19]. Among the diverse ecological functions performed by rhizosphere microorganisms, microbial carbon metabolism is an important component of plant–soil–microbe interactions [20]. Soil microorganisms regulate the transformation and utilization of plant-derived carbon through glycolysis, gluconeogenesis, the tricarboxylic acid (TCA) cycle, and various fermentation pathways [21]. These processes drive nutrient turnover, energy flow, and ecosystem functioning while simultaneously influencing soil fertility and nutrient availability [22]. Recent studies have shown that microbial carbon metabolism plays a crucial role in determining the fate of plant-fixed carbon and regulating nutrient acquisition by plants [23,24]. Differences in microbial carbon-metabolic potential may be associated with variation in nutrient turnover, plant physiological status, and crop quality [25]. Despite the recognized importance of carbon cycling in agroecosystems, whether propagation histories are associated with differences in rhizosphere microbial carbon-metabolic potential and tea quality remains insufficiently understood.

Recent advances in metabolomics and metagenomics have enabled integrated assessments of the relationships among soil nutrient status, rhizosphere microbial communities, microbial functional potential, and plant metabolic profiles [26,27,28,29]. These approaches provide an effective means of characterizing propagation-related differences across multiple biological levels. However, whether sexual and asexual propagation are associated with coordinated differences in rhizosphere microbial community composition, microbial carbon-metabolic potential, and leaf quality-related metabolic profiles remains unclear [30]. Therefore, sexually propagated (SR) and asexually propagated (AR) tea plants were comparatively investigated using an integrated approach combining soil physicochemical analyses, leaf physiological assessments, widely targeted metabolomics, and rhizosphere metagenomic profiling. The objectives of this study were to characterize the associations between propagation history and soil nutrient availability, rhizosphere microbial community composition, microbial carbon-cycling potential, and leaf metabolic profiles, and to examine these relationships from an integrated soil–microbiome–metabolome perspective in relation to propagation-associated variation in tea quality.

## 2. Materials and Methods

### 2.1. Experimental Site

The study was initiated in October 2022, and samples were collected during the spring tea-growing season of 2024 at the Tea Experimental Station of the Jiangxi Academy of Economic Crops, Jiangxi Province, China (28°22′ N, 116°00′ E). The experimental site is located in a major tea-producing region of southern China and has a typical subtropical monsoon climate characterized by abundant precipitation, moderate temperatures, and a long frost-free period.

The study was conducted in two immediately adjacent tea plantations established within the same experimental field in 1985 consisting of approximately 40-year-old tea plants (*Camellia sinensis* cv. ‘Fudingdabai’). Both the sexually propagated and asexually propagated plantations were established in 1985 and had since been maintained under comparable conventional agronomic practices, including fertilization, irrigation, annual light pruning, and pest and disease control. Each propagation type comprised three replicate plots, with approximately 200 tea plants per plot and 600 plants in total. Based on a row spacing of 1.5 m and an intra-row spacing of approximately 0.20 m, the net planted area of each replicate plot was estimated to be approximately 60 m^2^, corresponding to approximately 180 m^2^ for each propagation type and 360 m^2^ for the entire experimental area. According to records from the experimental station, the sexually propagated plantation was established using seedlings raised from naturally produced, open-pollinated seeds, whereas the asexually propagated plantation was established using rooted stem cuttings obtained from selected mother plants of the same cultivar. Both types of planting material were initially raised in a nursery and subsequently transplanted into the experimental field.

The soil at the experimental site was classified as red soil derived from Quaternary red clay. Before the tea plantations were established in 1985, composite bulk soil samples were collected from the 0–20 cm soil layer across the experimental site to characterize the original soil physicochemical properties. The soil was strongly acidic, with a pH of 4.48. Soil organic matter (SOM), total nitrogen (TN), total phosphorus (TP), and total potassium (TK) contents were 31.04, 2.59, 2.19, and 4.21 g kg^−1^, respectively. The alkali-hydrolyzable nitrogen (AN), available phosphorus (AP), and available potassium (AK) contents were 130.71, 115.34, and 158.25 mg kg^−1^, respectively.

### 2.2. Experimental Design and Sampling

Sexually propagated tea plants (SR) and asexually propagated tea plants (AR) of the cultivar ‘Fudingdabai’ were used as the experimental materials. The SR plantation was established using seedlings raised from naturally produced, open-pollinated seeds collected from ‘Fudingdabai’ tea plants, whereas the AR plantation was established using rooted stem cuttings obtained from selected mother plants of the same cultivar. To minimize the influence of factors other than propagation method, both plantations were located at the same experimental station and managed under identical agronomic practices, including fertilization, pruning, irrigation, and pest and disease control.

A comparative field design was used to evaluate differences between SR and AR tea plants. Each propagation type comprised three spatially independent replicate plots. Within each replicate plot, three to five tea plants with similar canopy development, growth status, and developmental stage and without visible symptoms of pests, diseases, or mechanical damage were randomly selected for sampling. Sampling was conducted during the spring tea-growing season of 2024, when the tea plants had reached the one-bud-and-two-adjacent-leaves stage. Fresh leaves consisting of one bud and two adjacent leaves were collected from the selected tea plants within each replicate plot and pooled to form one composite biological sample. The fresh leaf samples were immediately frozen in liquid nitrogen after collection, transported to the laboratory under frozen conditions, and divided into two portions. One portion was used for the determination of tea quality-related traits and physiological parameters, whereas the other portion was stored at −80 °C for subsequent metabolomic analysis.

Rhizosphere soil samples were collected simultaneously from the same tea plants used for leaf sampling. Briefly, the selected tea plants were carefully excavated, and loosely attached soil was removed by gentle shaking. The soil adhering to the root surfaces was collected using sterile brushes. Within each replicate plot, rhizosphere soil collected from three to five tea plants was thoroughly mixed to obtain one composite biological sample. Approximately 50–100 g of rhizosphere soil was collected for each composite sample. After removing roots and plant residues through a 2 mm sieve, each sample was divided into two portions. One portion was air-dried for soil physicochemical analysis, whereas the other portion was immediately frozen in liquid nitrogen and stored at −80 °C for metagenomic sequencing and functional analysis.

### 2.3. Soil Physicochemical Analysis

Air-dried rhizosphere soil samples were used for the determination of soil physicochemical properties. Soil pH was measured potentiometrically using a pH meter (PHS-3C, INESA Scientific Instrument Co., Ltd., Shanghai, China) at a soil-to-water ratio of 1:2.5 (*w*/*v*), as described by Lu [31]. Soil organic matter (SOM) was determined using the potassium dichromate oxidation method [31]. Total nitrogen (TN) was measured using the Kjeldahl digestion method according to Wang et al. [32]. Total phosphorus (TP) was quantified using the molybdenum–antimony colorimetric method after acid digestion, whereas total potassium (TK) was measured by flame photometry [31]. Alkali-hydrolyzable nitrogen (AN) was determined using the alkali diffusion method [31]. Available phosphorus (AP) was extracted with 0.5 M NaHCO_3_ and quantified according to He et al. [33], whereas available potassium (AK) was extracted with 1 M ammonium acetate and measured by flame photometry [31]. All measurements were conducted using three biological replicates.

### 2.4. Analysis of Tea Quality Components

Dried tea samples were used for the determination of quality-related chemical components. Tea polyphenol content was determined using the Folin–Ciocalteu colorimetric method according to Mao et al. [34]. Briefly, tea polyphenols were extracted from ground tea samples with 70% methanol, and the absorbance was measured at 765 nm using gallic acid as the calibration standard. Free amino acid content was determined using the ninhydrin colorimetric method as described by Ma et al. [35]. Water extract content was determined according to Tang et al. [36]. Briefly, 2.0 g of ground tea sample was extracted with 300 mL of boiling distilled water for 45 min. The extract was filtered through qualitative filter paper, and an aliquot of the filtrate was evaporated to dryness and weighed to determine the water extract content. All measurements were conducted using three biological replicates.

### 2.5. Analysis of Reactive Oxygen Species and Enzyme Activities

Fresh leaf samples (0.5 g) from each composite biological sample were homogenized in 10 mL of pre-cooled phosphate buffer (pH 7.0) and centrifuged at 12,000× *g* for 20 min at 4 °C. The resulting supernatant was used for the determination of reactive oxygen species levels and antioxidant enzyme activities.

The hydrogen peroxide (H_2_O_2_) content, superoxide anion (O_2_^−^) production rate, and activities of superoxide dismutase (SOD), peroxidase (POD), catalase (CAT), polyphenol oxidase (PPO), and indole-3-acetic acid (IAA) oxidase were determined using commercial assay kits obtained from Nanjing Jiancheng Bioengineering Institute (Nanjing, China). The catalogue numbers of the H_2_O_2_, O_2_^−^, SOD, POD, CAT, PPO, and IAA oxidase assay kits were A064-1-1, A052-1-1, A001-3-1, A084-3-1, A007-1-1, A136-1-1, and A078-1-1, respectively. All assay kits were stored at 2–8 °C before use, and measurements were performed strictly according to the manufacturer’s instructions.

Absorbance changes were monitored using a UV–Vis spectrophotometer (UV-2600, Shimadzu Corporation, Kyoto, Japan), and enzyme activities were calculated according to the protocols provided by the manufacturer. All measurements were conducted using three biological replicates.

### 2.6. Widely Targeted Metabolomic Analysis

Fresh leaf samples consisting of one bud and two adjacent leaves were collected from multiple tea plants within each biological replicate and pooled to form one composite sample, as described in Section 2.2. The same samples were used for metabolomic analysis. Widely targeted metabolomic profiling was performed using an ultra-performance liquid chromatography–tandem mass spectrometry (UPLC–MS/MS) platform consisting of a Shimadzu UPLC system (Shimadzu Corporation, Kyoto, Japan) coupled with a QTRAP 6500+ mass spectrometer (AB Sciex, Framingham, MA, USA).

The samples were freeze-dried and ground into a fine powder using a mixer mill with zirconia beads. Approximately 50 mg of powdered sample was extracted with 1.2 mL of pre-cooled 70% methanol. The mixture was vortexed for 30 s at 30 min intervals for a total of six cycles and then incubated overnight at 4 °C. After centrifugation at 12,000 rpm for 3 min, the supernatant was collected and filtered through a 0.22 μm membrane before UPLC–MS/MS analysis. Metabolites were detected in multiple reaction monitoring (MRM) mode. Metabolite identification was performed by matching retention times and MS/MS spectra against an in-house metabolite database maintained by Metware Biotechnology Co., Ltd. (Wuhan, China) and relevant public databases. Relative metabolite abundance was quantified based on MRM signal intensity. Quality-control samples were prepared by pooling equal aliquots from all biological samples and were analyzed periodically throughout the analytical sequence to assess instrument stability and analytical reproducibility.

Raw metabolomic data were normalized before statistical analysis. Principal component analysis (PCA) was performed to evaluate overall metabolic variation among samples, whereas orthogonal partial least squares discriminant analysis (OPLS-DA) was used to identify metabolites contributing to group separation. Differentially accumulated metabolites (DAMs) were screened using a VIP value ≥1 and an absolute log_2_ fold change (|log_2_FC|) ≥ 1. Volcano plots, hierarchical clustering heatmaps, and Kyoto Encyclopedia of Genes and Genomes (KEGG) enrichment analyses were performed to visualize metabolite differences and identify significantly enriched metabolic pathways. All metabolomic analyses were conducted by Metware Biotechnology Co., Ltd. (Wuhan, China).

### 2.7. DNA Extraction and Metagenomic Sequencing

Total microbial DNA was extracted from rhizosphere soil samples using a commercial soil DNA extraction kit according to the manufacturer’s instructions. DNA concentration and purity were assessed using a NanoDrop 2000 spectrophotometer (Thermo Fisher Scientific, Wilmington, DE, USA), and DNA integrity was evaluated by agarose gel electrophoresis.

Sequencing libraries were constructed and sequenced on the Illumina NovaSeq 6000 platform (Illumina Inc., San Diego, CA, USA) using paired-end sequencing. Raw reads were quality-filtered by removing adapters, low-quality sequences, and host-derived reads. Clean reads were assembled into contigs using MEGAHIT version 1.2.9. Open reading frames (ORFs) were predicted using Prodigal version 2.6.3 to generate a non-redundant gene catalog for subsequent taxonomic and functional annotation analyses.

### 2.8. Taxonomic Annotation and Microbial Community Structure Analysis

Taxonomic annotation of predicted genes was performed using DIAMOND version 2.1.9.163 against the NCBI non-redundant (NR) protein database (version 2022.05), which included bacterial, fungal, archaeal, and viral sequences. DIAMOND blastp searches were conducted with an e-value threshold of 1 × 10^−5^ [37]. Taxonomic assignments were determined using the lowest common ancestor (LCA) algorithm implemented in MEGAN (https://software-ab.cs.uni-tuebingen.de/download/megan6/welcome.html; accessed on 1 August 2026). Relative abundances of microbial taxa at different taxonomic levels were calculated based on the summed abundances of genes assigned to each taxon.

Differences in microbial community composition between the SR and AR groups were evaluated using principal coordinate analysis (PCoA) based on Bray–Curtis dissimilarities, implemented using the stats package version 4.2.0. Taxonomic composition at different classification levels was visualized using ggplot2 version 3.3.6. Differentially abundant taxa between the SR and AR groups were identified using LEfSe version 1.1.2 [38] and were subsequently included in the correlation analyses.

### 2.9. Functional Annotation and Carbon-Cycling Pathway Analysis

Functional annotation of non-redundant genes was performed against the Kyoto Encyclopedia of Genes and Genomes (KEGG) database based on annotated KEGG Orthology (KO) entries. To evaluate differences in microbial metabolic potential associated with propagation methods, genes involved in central carbon metabolism were extracted, including glycolysis, gluconeogenesis, the tricarboxylic acid (TCA) cycle, pyruvate metabolism, and fermentation-related pathways.

The relative abundances of carbon metabolism pathways were calculated and compared between SR and AR treatments. Associations between carbon metabolic pathways and dominant microbial taxa were evaluated using Spearman’s rank correlation analysis in R (version 4.3.1; R Foundation for Statistical Computing, Vienna, Austria) based on the relative abundances of pathways and microbial taxa. Significant associations with |r| > 0.7 and *p* < 0.05 were retained for network construction. The resulting correlation matrix was visualized and analyzed using Cytoscape (version 3.9.1, Cytoscape Consortium, San Diego, CA, USA) [39].

### 2.10. Microbiota–Metabolite Correlation Analysis

To evaluate the associations between rhizosphere microbial communities and leaf metabolic profiles, Spearman’s rank correlation analysis was performed between enriched microbial taxa and differentially accumulated metabolites (DAMs). Correlations were calculated in R (version 4.3.1; R Foundation for Statistical Computing, Vienna, Austria) using the relative abundances of microbial taxa and normalized abundances of DAMs. Significant correlations with |*r*| > 0.7 and *p* < 0.05 were retained for visualization. Correlation heatmaps were generated to illustrate the relationships between microbial taxa and metabolites.

### 2.11. Statistical Analysis

All data are presented as mean ± standard error (SE). Differences between SR and AR tea plants were evaluated using independent-samples Student’s *t*-tests, with statistical significance defined as *p* < 0.05.

Principal component analysis (PCA) and orthogonal partial least squares discriminant analysis (OPLS-DA) were performed to assess overall metabolic variation and sample discrimination. Principal coordinate analysis (PCoA) based on Bray–Curtis dissimilarity was conducted to evaluate differences in microbial community structure and functional composition between treatments. Differentially accumulated metabolites (DAMs) were identified based on a VIP ≥ 1 and |log_2_FC| ≥ 1. Hierarchical clustering, volcano plots, and pathway enrichment analyses were performed to characterize metabolite variation patterns. Differences in the relative abundances of KEGG functional pathways between SR and AR were assessed using independent-samples Student’s *t*-tests.

All statistical analyses were performed using SPSS 26.0 (IBM Corp., Armonk, NY, USA). Data visualization was conducted using Origin 2024 (OriginLab Corporation, Northampton, MA, USA) and R software (version 4.3.1).

## 3. Results

### 3.1. Soil Properties and Leaf Physiological Traits

**Figure 1:**
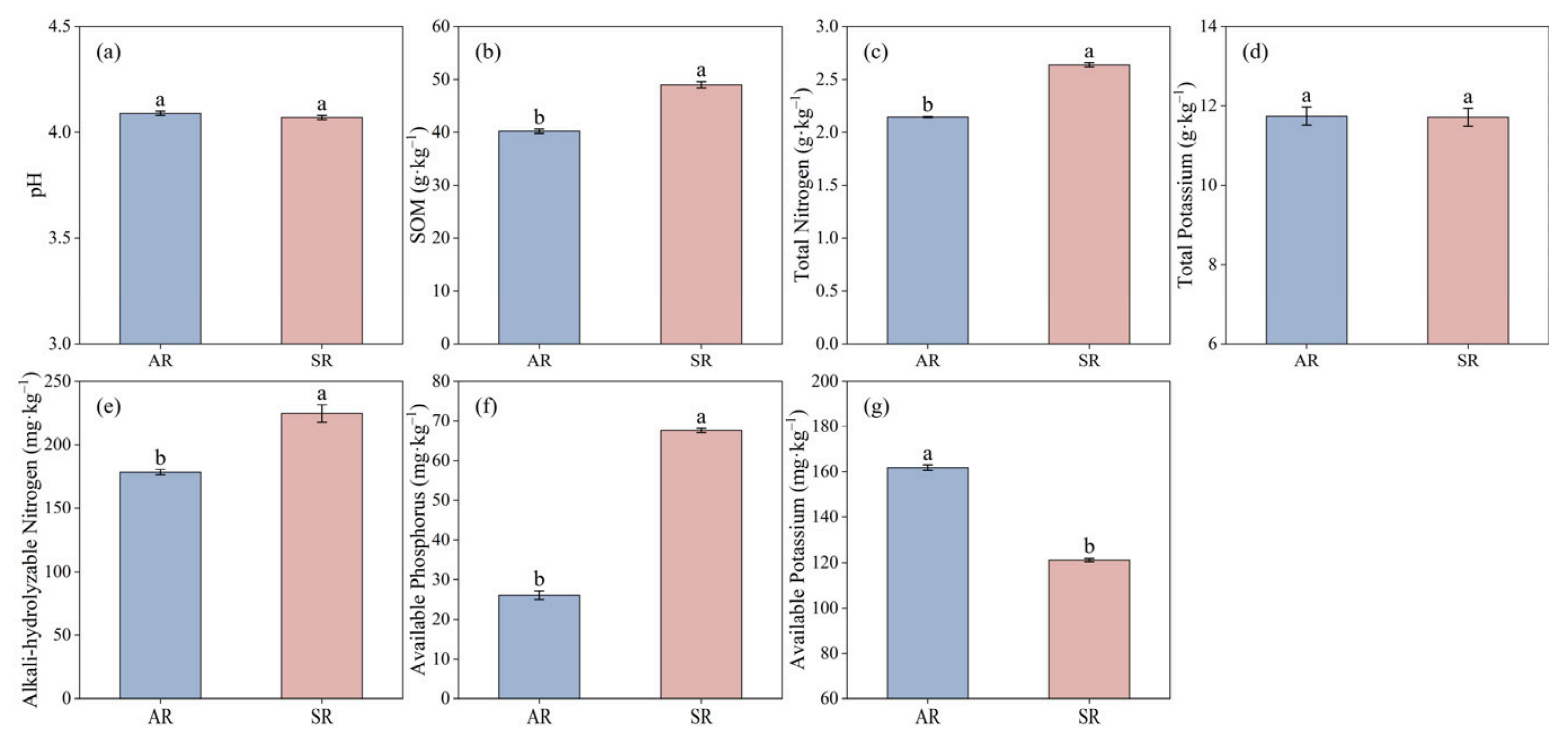
Variations in rhizosphere soil physicochemical properties between SR and AR tea plantations. (**a**) Soil pH; (**b**) soil organic matter (SOM); (**c**) total nitrogen; (**d**) total potassium; (**e**) alkali-hydrolyzable nitrogen; (**f**) available phosphorus; and (**g**) available potassium. Note: SR and AR represent sexually and asexually propagated tea plants, respectively. Data are expressed as mean ± standard error (SE) (*n* = 3). Different lowercase letters indicate significant differences between treatments at *p* < 0.05.

The physicochemical properties of rhizosphere soil were compared between SR and AR tea plantations (Figure 1). Soil pH and total potassium (TK) did not differ significantly between SR and AR tea plantations. Compared with AR, SR tea plantations exhibited significantly higher soil organic matter (SOM), total nitrogen (TN), alkali-hydrolyzable nitrogen (AN), and available phosphorus (AP) contents by 23.1%, 18.2%, 27.8%, and 161.5%, respectively (*p* < 0.05). In contrast, available potassium (AK) content was significantly lower in SR than in AR by 24.4% (*p* < 0.05). These results indicate that SR and AR plantations differed in rhizosphere soil nutrient characteristics, with SR showing greater SOM accumulation and higher nitrogen and phosphorus availability.

### 3.2. Leaf Quality-Related Traits and Physiological Responses

**Figure 2:**
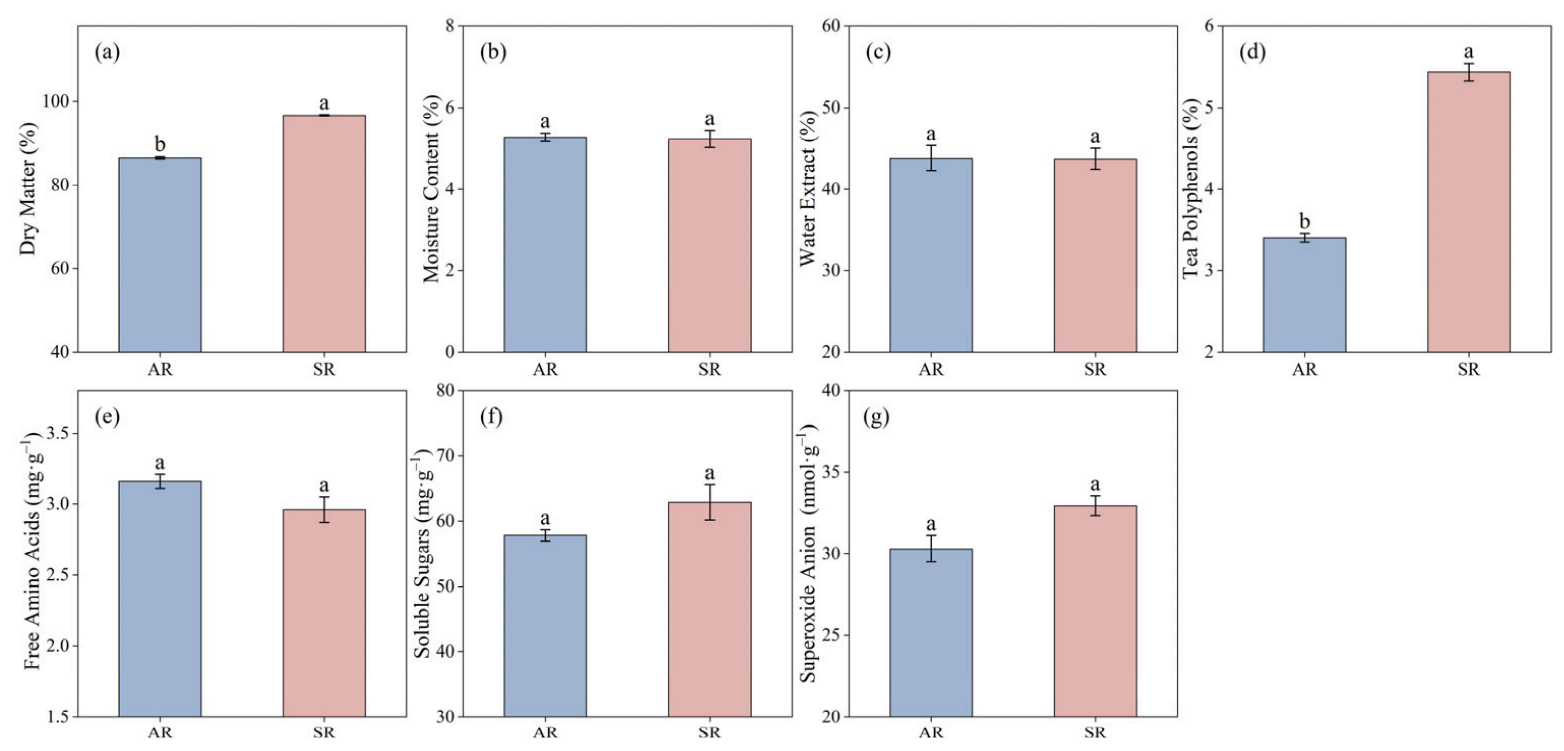
Variations in quality-related traits of fresh tea leaves between SR and AR tea plants. (**a**) Dry matter; (**b**) moisture content; (**c**) water extract; (**d**) tea polyphenols; (**e**) free amino acids; (**f**) soluble sugars; and (**g**) superoxide anion content. Note: SR and AR represent sexually and asexually propagated tea plants, respectively. Data are expressed as mean ± standard error (SE) (*n* = 3). Different lowercase letters indicate significant differences between treatments at *p* < 0.05.

Fresh leaves exhibited significant differences in several quality-related traits between SR and AR tea plants (Figure 2). Moisture content, water extract content, and free amino acid content showed no significant differences between treatments. Compared with AR, SR exhibited significantly higher dry matter, tea polyphenol, and soluble sugar contents by 11.5%, 58.8%, and 8.6%, respectively (*p* < 0.05). The superoxide anion (O_2_^−^) production rate was also significantly higher in SR than in AR, with an increase of 9.3% (*p* < 0.05). These results indicate that SR and AR tea plants differed in quality-related components, with SR showing higher accumulation of tea polyphenols and soluble sugars.

**Figure 3:**
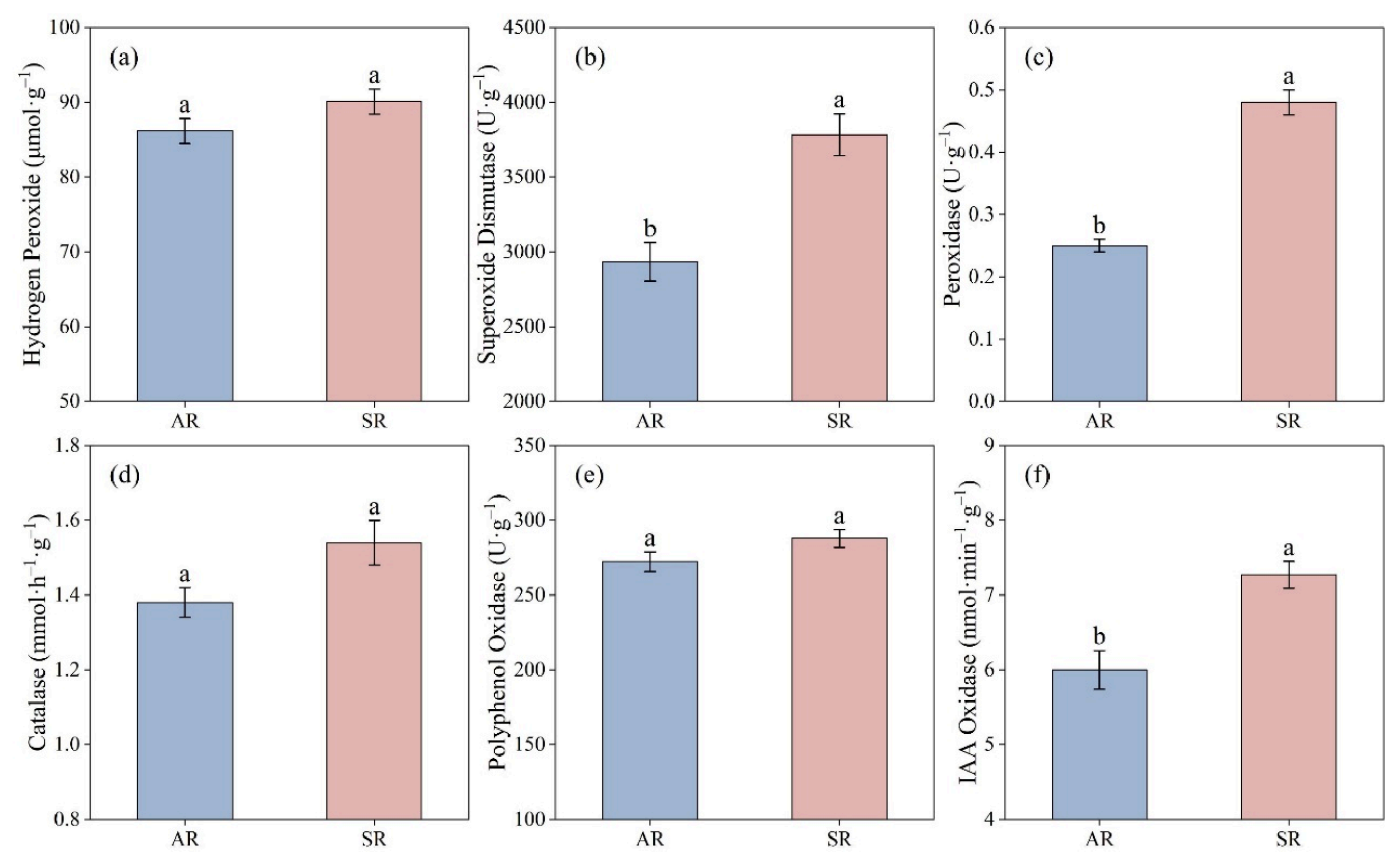
Reactive oxygen species levels and enzyme activities in tea leaves of SR and AR tea plants. (**a**) Hydrogen peroxide content; (**b**) superoxide dismutase activity; (**c**) peroxidase activity; (**d**) catalase activity; (**e**) polyphenol oxidase activity; and (**f**) indole-3-acetic acid (IAA) oxidase activity. Note: SR and AR represent sexually and asexually propagated tea plants, respectively. Data are expressed as mean ± standard error (SE) (*n* = 3). Different lowercase letters indicate significant differences between treatments at *p* < 0.05. The contents of quality-related components were expressed on a dry weight basis.

Reactive oxygen species levels and antioxidant enzyme activities also differed between propagation methods (Figure 3). H_2_O_2_ content, catalase (CAT) activity, and polyphenol oxidase (PPO) activity showed no significant differences between SR and AR. In contrast, superoxide dismutase (SOD), peroxidase (POD), and indole-3-acetic acid (IAA) oxidase activities were significantly increased under SR by 30.4%, 92.0%, and 21.7%, respectively (*p* < 0.05). Overall, SR exhibited higher activities of antioxidant-related enzymes while maintaining H_2_O_2_ homeostasis.

### 3.3. Metabolomic Profiling and Differential Metabolite Accumulation

**Figure 4:**
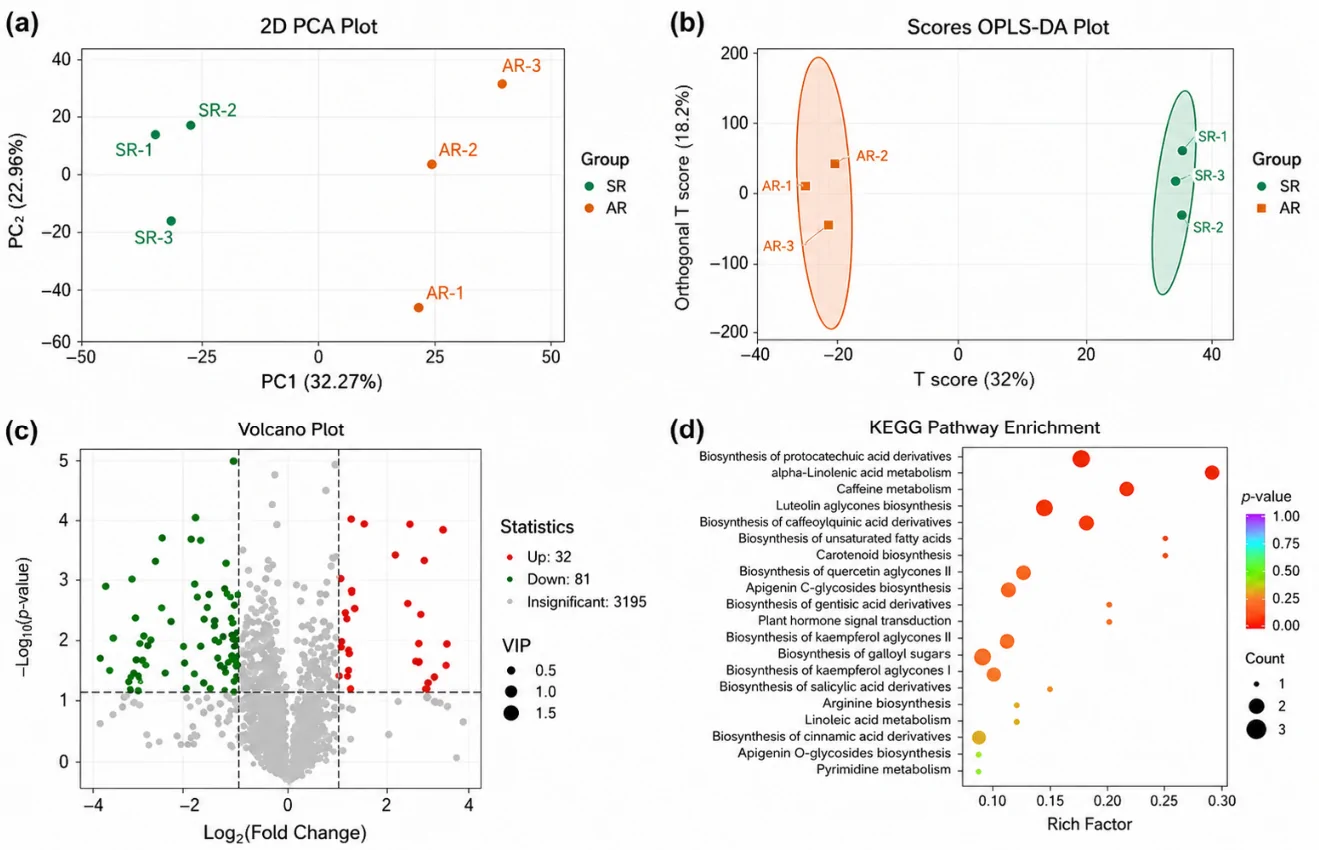
Metabolic profiling and pathway enrichment analysis of differentially accumulated metabolites in tea leaves between AR and SR tea plants. PCA (**a**), OPLS-DA (**b**), volcano plot of differentially accumulated metabolites (**c**), and KEGG pathway enrichment analysis (**d**). Note: Each point in PCA and OPLS-DA plots represents an independent biological replicate. Red and green dots in the volcano plot indicate significantly upregulated and downregulated metabolites, respectively, while gray dots represent non-significant metabolites. In the KEGG enrichment plot, bubble size indicates the number of enriched metabolites and color represents the significance level of enrichment.

Widely targeted metabolomic profiling of tea leaves revealed clear differences in metabolic composition between SR and AR tea plants (Figure 4). Both PCA and OPLS-DA showed distinct separation between the two treatments, indicating substantial metabolic divergence. A total of 1248 metabolites were identified across all samples using the widely targeted metabolomics platform. Among them, 113 differentially accumulated metabolites (32 upregulated and 81 downregulated) were identified between SR and AR. KEGG enrichment analysis showed that these metabolites were mainly involved in α-linolenic acid metabolism, caffeine metabolism, carotenoid biosynthesis, plant hormone signal transduction, and flavonoid-related biosynthetic pathways.

**Figure 5:**
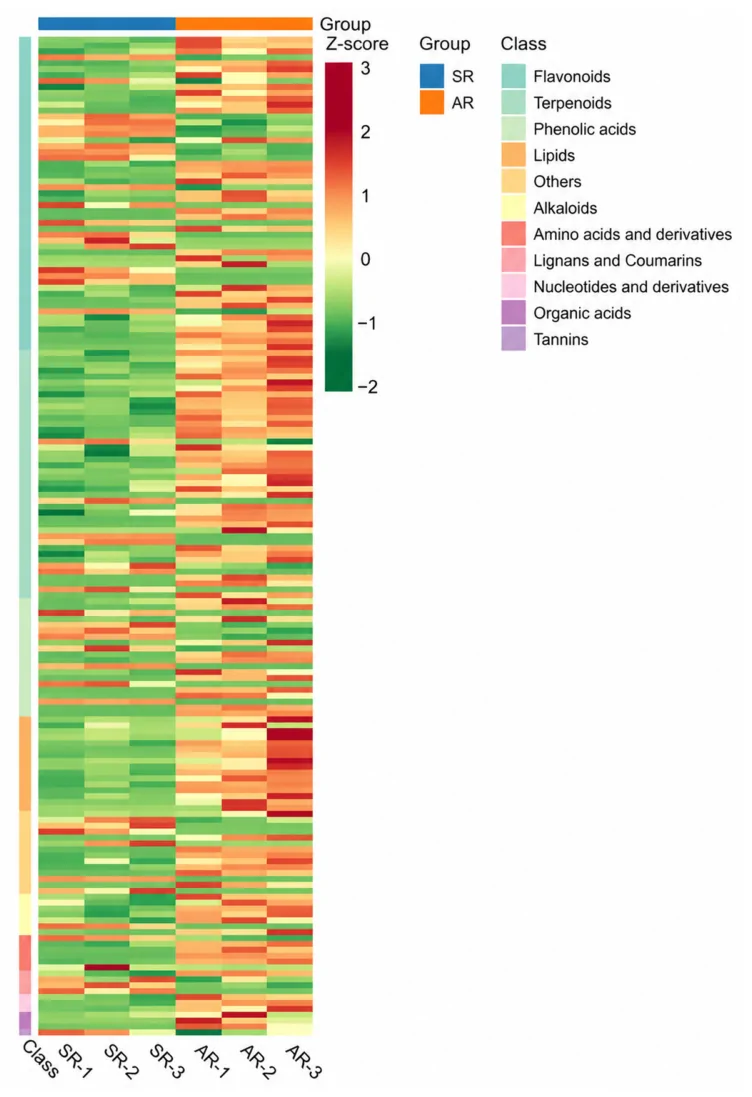
Heatmap visualization of differential metabolite accumulation patterns between SR and AR tea plants. Note: Rows represent differential metabolites and columns represent biological replicates. Colors indicate normalized metabolite abundance based on Z-score transformation, with red and green representing relatively high and low accumulation levels, respectively. Metabolites are classified into flavonoids, terpenoids, phenolic acids, lipids, alkaloids, amino acids and derivatives, lignans and coumarins, nucleotides and derivatives, organic acids, and tannins.

The heatmap further confirmed distinct metabolite accumulation patterns between SR and AR (Figure 5). Biological replicates within each treatment clustered closely, indicating good reproducibility of the metabolomic profiles. Differential metabolites mainly included flavonoids, phenolic acids, lipids, terpenoids, alkaloids, amino acids and derivatives, lignans and coumarins, nucleotides and derivatives, organic acids, and tannins. These results indicate distinct leaf metabolic profiles between SR and AR tea plants, particularly in metabolites associated with secondary metabolic pathways.

### 3.4. Rhizosphere Microbial Community Structure and Biomarkers

**Figure 6:**
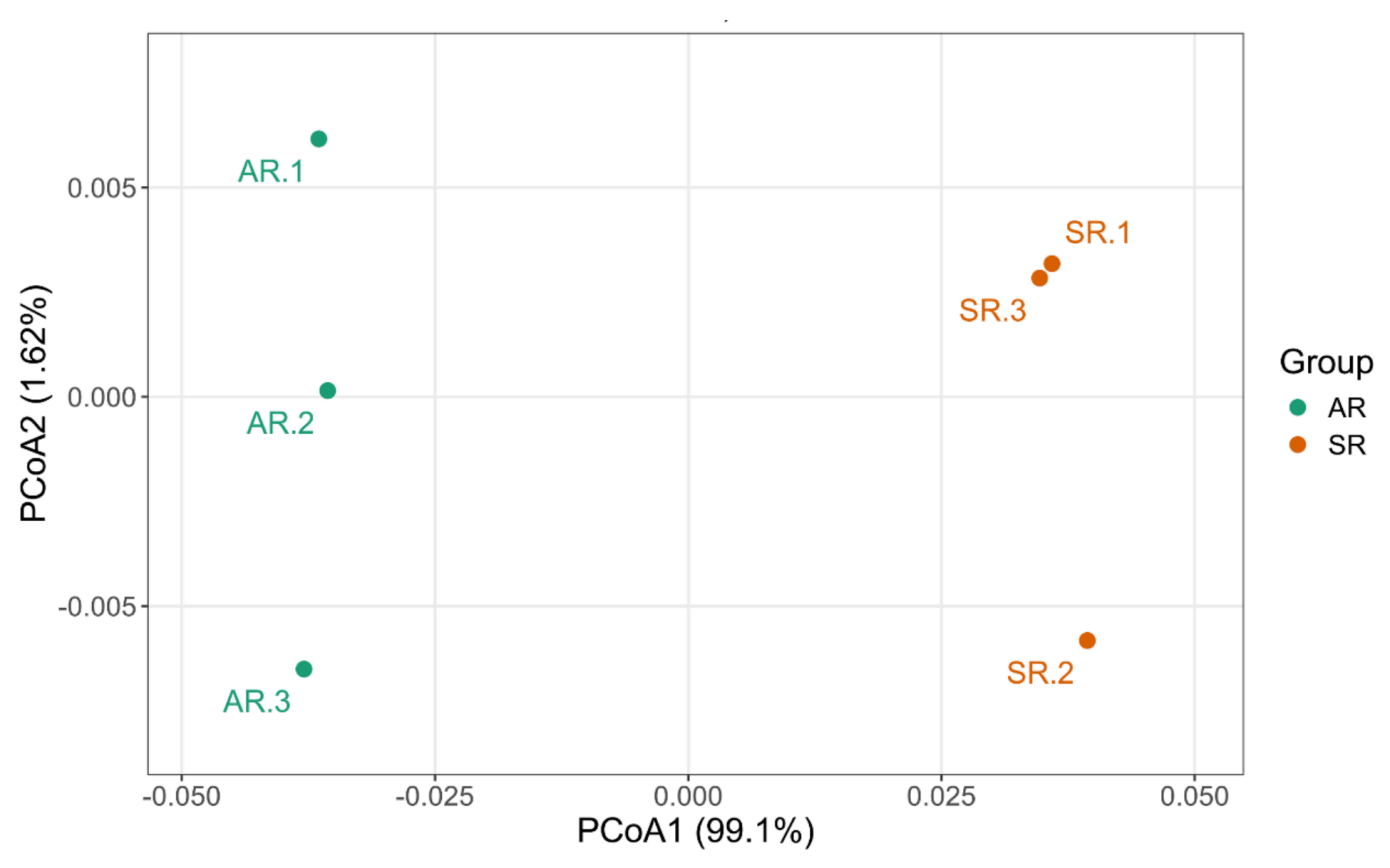
Principal coordinate analysis (PCoA) of rhizosphere microbial community composition in SR and AR tea plants. Note: SR and AR represent sexually propagated and asexually propagated tea plants, respectively. Each point represents an independent biological replicate (*n* = 3). PCoA was conducted based on Bray–Curtis dissimilarity matrices to evaluate differences in rhizosphere microbial community composition between SR and AR tea plants.

Rhizosphere microbial community composition differed markedly between SR and AR (Figure 6). PCoA based on Bray–Curtis distances showed clear separation between the two treatments, with PCoA1 and PCoA2 explaining 99.1% and 1.62% of the total variation, respectively. Samples within each treatment clustered closely, indicating high reproducibility among biological replicates. These results indicate distinct rhizosphere microbial community structures between SR and AR tea plants.

**Figure 7:**
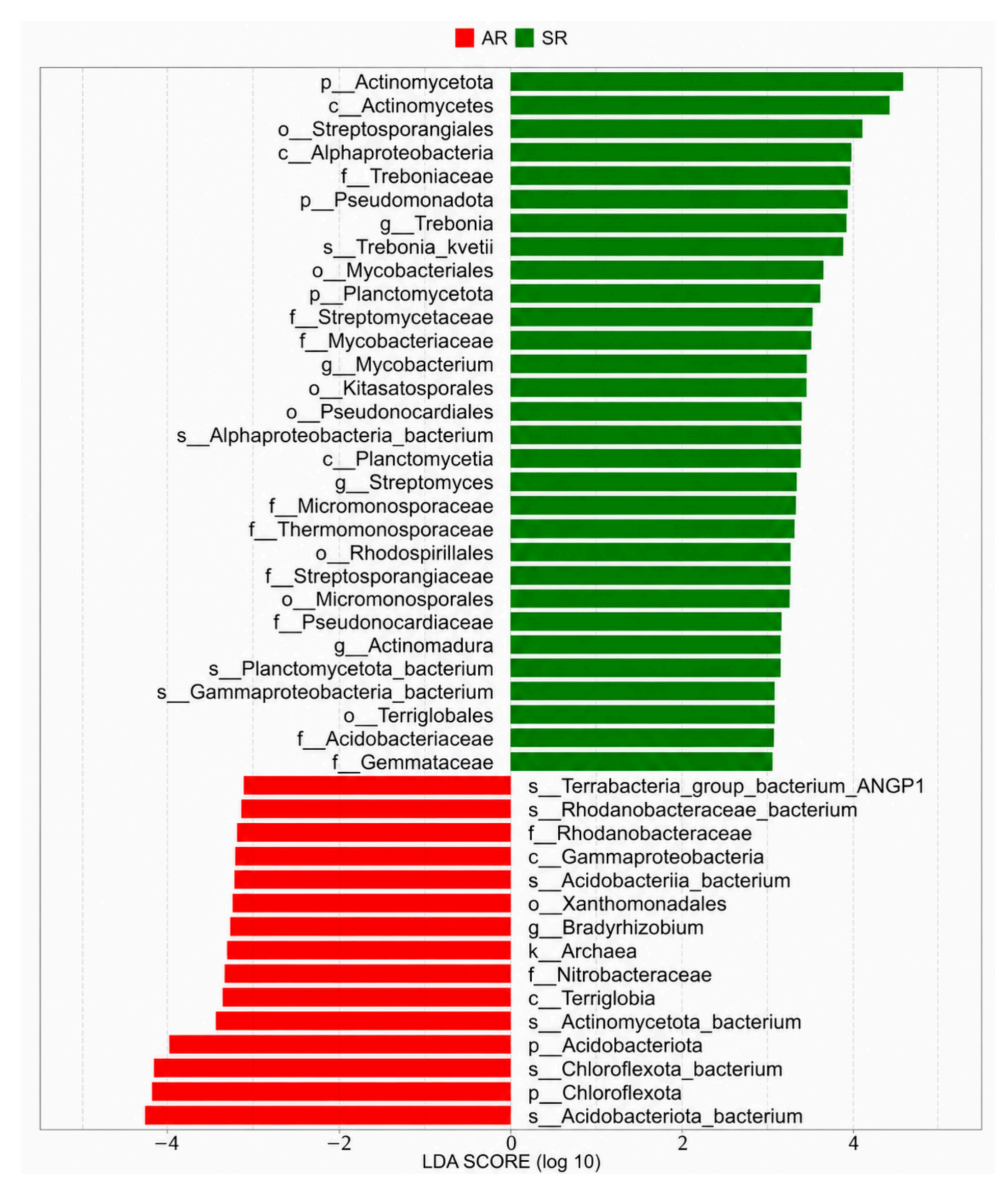
LEfSe analysis identifying differentially abundant rhizosphere microbial taxa between SR and AR tea plants. Note: Green and red bars indicate taxa significantly enriched in SR and AR, respectively. The length of each bar represents the logarithmic LDA score, indicating the contribution of each taxon to the discrimination between groups. Taxa were selected based on LDA score thresholds and statistical significance.

LEfSe analysis identified microbial taxa that contributed to the differentiation between SR and AR rhizosphere communities (Figure 7). SR was enriched with Actinomycetota, Alphaproteobacteria, Streptomycetales, Pseudomonadota, and Planctomycetota, whereas AR showed higher abundances of Acidobacteriota, Chloroflexota, *Bradyrhizobium*, Nitrobacteraceae, and several archaeal lineages. The SR-enriched taxa were mainly associated with nutrient transformation and organic matter turnover, whereas AR-enriched taxa were primarily related to soil nutrient cycling and stress-adaptation processes.

### 3.5. Microbial Functional Profiles and Carbon-Cycling Pathways

**Figure 8:**
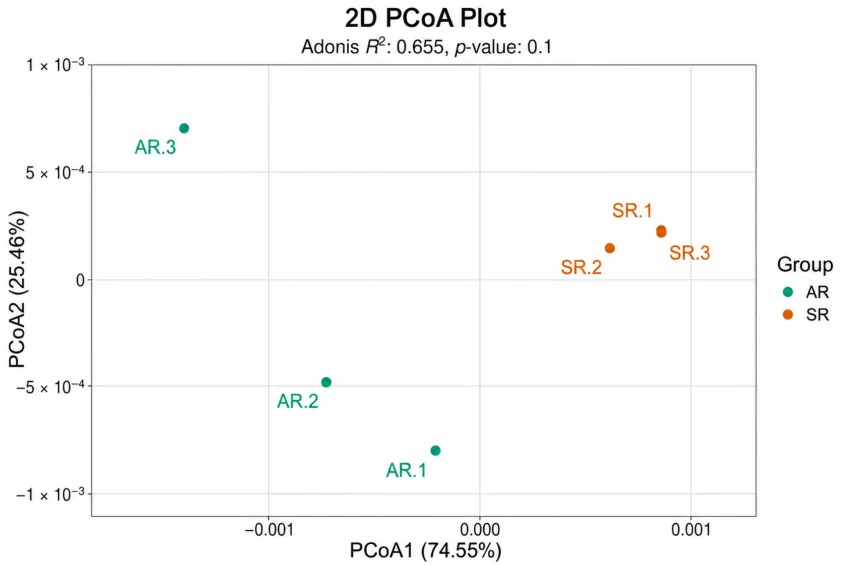
Principal coordinate analysis (PCoA) of rhizosphere microbial functional profiles in SR and AR tea plants. Note: Each point represents an independent biological replicate. PCoA was performed based on Bray–Curtis distances calculated from microbial functional profiles. PCoA1 and PCoA2 explained 74.50% and 25.46% of the total variation, respectively.

Functional annotation of rhizosphere metagenomic data revealed distinct microbial functional profiles between SR and AR tea plants (Figure 8). PCoA based on functional composition showed clear separation between SR and AR, with PCoA1 and PCoA2 explaining 74.5% and 25.46% of the total variation, respectively. This separation indicates that rhizosphere microbial communities associated with SR and AR tea plants differed in both taxonomic composition and functional potential.

### 3.6. Microbiome–Metabolome Association and Co-Occurrence Network

**Figure 9:**
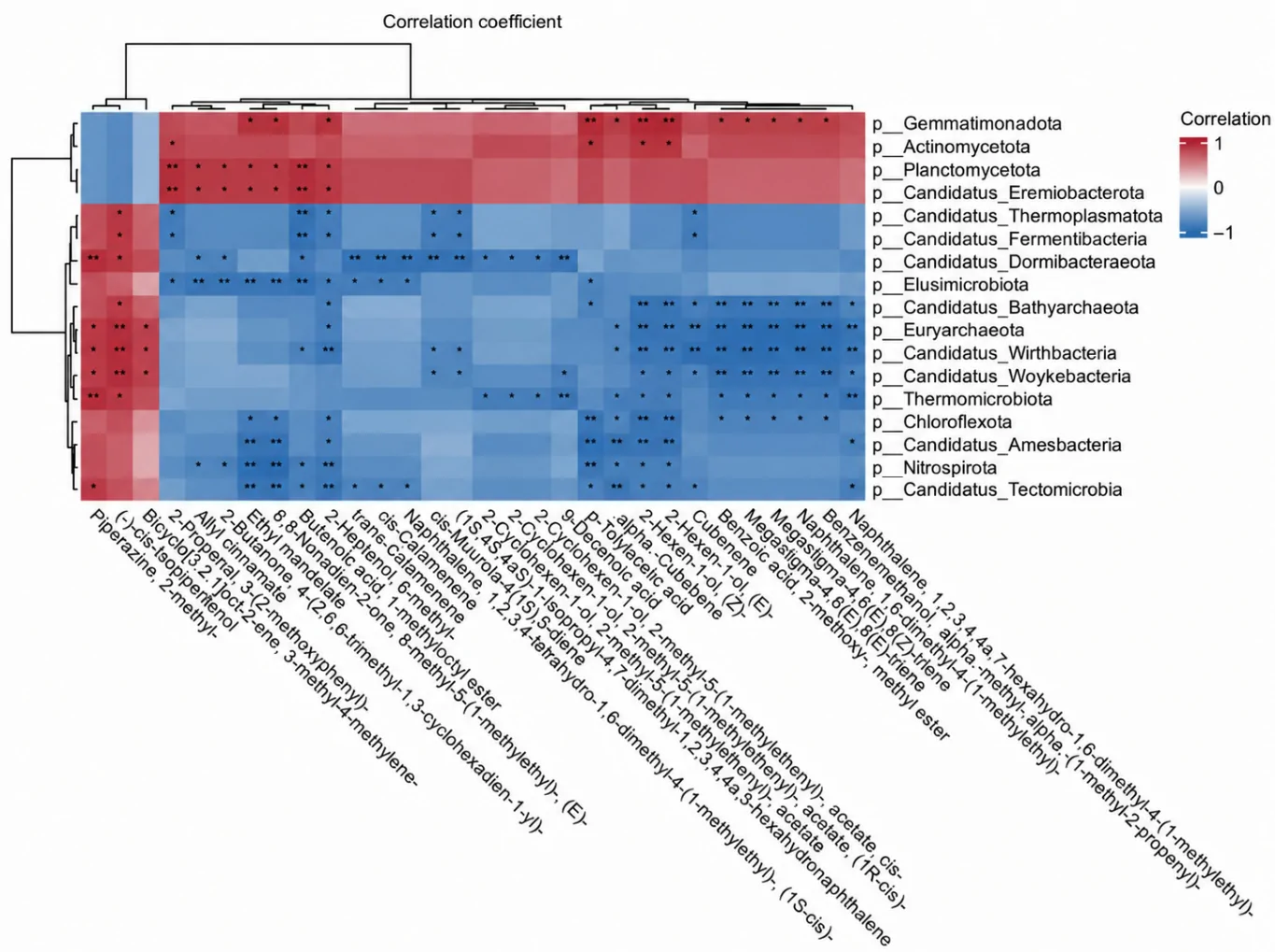
Hierarchical clustering heatmap of Spearman correlations between rhizosphere microbial phyla and differentially accumulated metabolites in tea leaves. Note: Rows represent microbial phyla identified from rhizosphere metagenomic samples, whereas columns represent differentially accumulated metabolites detected in tea leaf samples. Dendrograms indicate hierarchical clustering patterns of microbial taxa and metabolites. Red and blue colors indicate positive and negative correlations, respectively. Only significant correlations are shown (* *p* < 0.05; ** *p* < 0.01).

Correlation analysis integrating rhizosphere microbial taxa and leaf metabolites revealed extensive associations between dominant microbial taxa and differentially accumulated metabolites (Figure 9). Actinomycetota, Pseudomonadota, and Planctomycetota, which were enriched in SR, showed significant positive correlations with multiple flavonoids, phenolic acids, and soluble sugar-related metabolites. In contrast, Acidobacteriota and Chloroflexota, which were enriched in AR, were predominantly negatively correlated with these metabolites.

**Figure 10:**
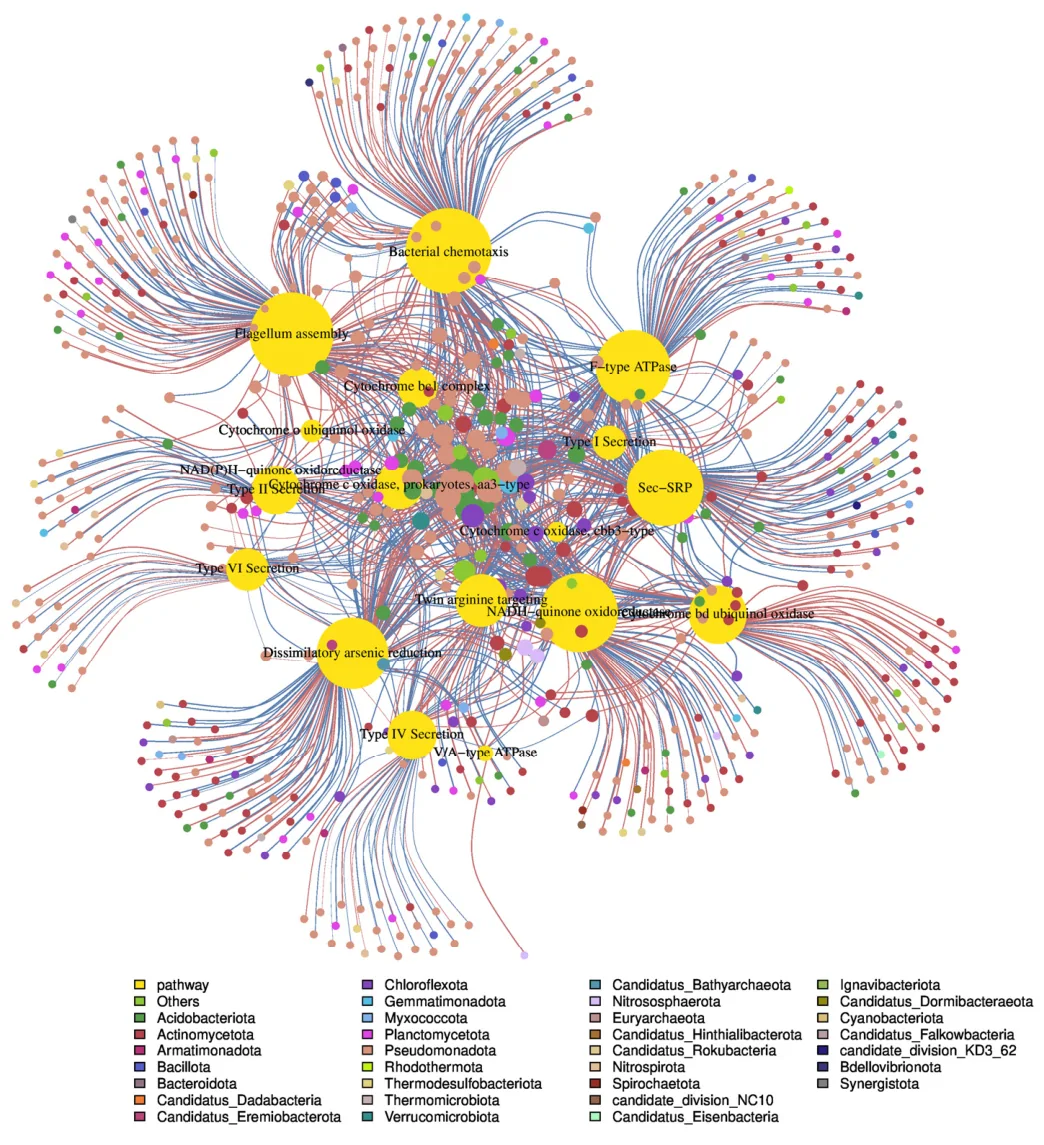
Co-occurrence network showing associations between microbial taxa and carbon-cycling pathways in rhizosphere soils of SR and AR tea plants. Note: Yellow nodes represent carbon-cycling pathways, whereas colored nodes represent microbial taxa. Node size is proportional to the connectivity degree, and edges indicate significant Spearman’s rank correlations between pathways and taxa (|*r*| > 0.7, *p* < 0.05). Cytoscape (version 3.9.1) was used exclusively for network visualization.

Co-occurrence network analysis identified significant associations between several dominant microbial taxa and carbon-cycling pathways, including the tricarboxylic acid cycle, glycolysis, gluconeogenesis, and acetate-producing fermentation pathways. The network was constructed from the combined dataset and was used to visualize taxon–pathway correlations rather than to compare network complexity between SR and AR. (Figure 10). Taxa affiliated with Actinomycetota, Pseudomonadota, Acidobacteriota, and Chloroflexota exhibited significant associations with carbon-cycling pathways.

## 4. Discussion

### 4.1. Differences in Soil Nutrient Status and Tea Quality-Related Traits Between SR and AR

Soil nutrient availability is an important factor associated with tea plant growth, productivity, and quality-related metabolite accumulation. Previous studies have shown that soil organic matter and available nutrient pools are closely related to nutrient supply capacity and the accumulation of quality-related compounds in tea plants [40,41]. In the present study, SR exhibited significantly higher SOM, TN, AN, and AP contents than AR, indicating distinct rhizosphere nutrient conditions between the two propagation types. Rhizosphere soil pH did not differ significantly between SR and AR despite the observed differences in nutrient availability. This pattern suggests that variation in nutrient status may be more closely associated with differences in soil organic matter accumulation and rhizosphere nutrient transformation processes than with soil pH alone. Similar patterns have been reported in perennial crops, where sexually propagated plants may develop more extensive root systems associated with differences in nutrient acquisition and soil resource utilization [42,43].

Nitrogen and phosphorus are closely related to tea quality formation because of their roles in amino acid metabolism, photosynthesis, energy transfer, and carbon metabolism [44,45]. Greater nitrogen and phosphorus availability may provide additional metabolic substrates and resources associated with the accumulation of flavonoids, phenolic compounds, and other quality-related metabolites [12]. In the present study, tea polyphenol and soluble sugar contents were significantly higher in SR than in AR, whereas free amino acid content did not differ significantly between the two groups. Although SR also exhibited higher soil nitrogen availability, free amino acid accumulation is influenced by multiple physiological processes, including nitrogen assimilation, amino acid metabolism, and carbon–nitrogen balance [14,35]. Therefore, the higher nutrient availability observed in SR may be more closely associated with the accumulation of tea polyphenols and soluble sugars than with variation in the free amino acid pool. Previous studies have similarly reported positive associations between soil organic matter, nitrogen and phosphorus availability, and the accumulation of tea polyphenols and soluble sugars [46,47]. The relatively large difference in tea polyphenol content between SR and AR further suggests that propagation-related variation in tea quality may involve additional long-term differences in plant growth characteristics and resource utilization patterns.

Plant physiological status may also be associated with variation in quality-related metabolite accumulation. Reactive oxygen species (ROS) function both as indicators of oxidative status and as signaling molecules involved in plant growth, development, and secondary metabolism [48]. Antioxidant enzymes, including SOD, POD, and CAT, contribute to cellular redox homeostasis [49]. In the present study, SR tea leaves exhibited higher SOD, POD, and IAA oxidase activities than AR tea leaves, whereas H_2_O_2_ content remained comparable between the two groups. Similar patterns have been reported in plants with differences in physiological activity and metabolic status [50]. These differences in antioxidant enzyme activities may be associated with variation in intracellular redox status and quality-related metabolite accumulation.

Collectively, SR and AR tea plants differed in rhizosphere nutrient status, physiological characteristics, and quality-related metabolite accumulation. The coordinated variation among these traits suggests potential associations between rhizosphere nutrient conditions, plant physiological status, and tea quality-related characteristics. Notably, soil available potassium (AK) was 24.4% lower in SR than in AR. This difference may be associated with variation in root development and potassium acquisition between propagation types [8], although plant potassium uptake was not directly measured in this study. Because SR still exhibited higher tea polyphenol and soluble sugar contents, the lower AK level did not appear to constrain these quality-related traits under the present field conditions. Long-term potassium balance nevertheless warrants further attention in sexually propagated tea plantations.

### 4.2. Differences in Rhizosphere Microbial Community Composition Between SR and AR

Our results revealed distinct rhizosphere microbial community structures between SR and AR tea plants. PCoA showed clear separation between the two groups. SR was characterized by higher relative abundances of Actinomycetota, Pseudomonadota, and Planctomycetota, whereas Acidobacteriota and Chloroflexota were more abundant in AR. These findings indicate differences in rhizosphere microbial community composition between tea plants with different propagation histories.

Rhizosphere microbial community composition is closely associated with host plant traits, including root architecture, nutrient acquisition patterns, and root exudate composition [51,52]. Previous studies have shown that variation in plant genetic background can be accompanied by differences in rhizosphere microbial community assembly [53,54]. Therefore, the microbial divergence observed in this study may be associated with differences in rhizosphere conditions between tea plants with different propagation histories. Further measurements of root architecture, nutrient acquisition, and root exudate composition would help clarify the ecological basis of these differences.

Actinomycetota, which were enriched in SR, are widely recognized for their roles in the degradation of complex organic substrates, soil organic matter turnover, and nutrient cycling [55]. Many members of this phylum are also associated with the production of plant growth-related compounds and antimicrobial metabolites [56]. Similarly, Pseudomonadota are generally regarded as copiotrophic microorganisms and are often associated with nutrient-rich environments and active carbon metabolism [57]. In the present study, the higher SOM, TN, and AP levels observed in SR coincided with greater relative abundances of these microbial groups, suggesting associations between rhizosphere nutrient conditions and microbial community composition. In contrast, Acidobacteriota enriched in AR are commonly considered oligotrophic microorganisms adapted to relatively nutrient-limited environments [58], while Chloroflexota are also frequently associated with ecological niches characterized by lower nutrient availability and slower metabolic turnover [59]. The greater relative abundances of these taxa in AR may therefore reflect differences in rhizosphere ecological conditions between the two propagation types.

Similar patterns have been reported in other perennial plant systems. Xiong et al. [60] reported that plants with greater nutrient-use efficiency tended to harbor more Actinomycetota and Pseudomonadota, whereas Acidobacteriota were more abundant in plants with lower nutrient acquisition capacity. Our observations are generally consistent with these findings. Unlike previous studies that mainly focused on cultivar differences or fertilization regimes, the present results suggest that rhizosphere microbial community structure differed between tea plants with different propagation histories under comparable long-term management conditions. Collectively, these patterns further support an association between propagation history and rhizosphere microbial community composition. PCoA1 explained 99.1% of the total variation, reflecting pronounced separation between SR and AR samples in the present dataset.

### 4.3. Differences in Rhizosphere Microbial Functional and Carbon-Cycling Profiles

Functional annotation revealed clear differences in rhizosphere microbial functional profiles between SR and AR tea plants. Functional PCoA showed distinct separation between the two groups, indicating variation in microbial metabolic potential associated with different propagation histories. Carbon-cycling functions, including glycolysis, gluconeogenesis, the tricarboxylic acid (TCA) cycle, and fermentation-related pathways, were prominent components of the microbial functional profiles. Differences in these functional characteristics suggest variation in microbial potential for carbon transformation and utilization between SR and AR rhizosphere communities.

Carbon cycling is a fundamental ecological process involved in organic matter transformation, energy flow, and nutrient turnover in terrestrial ecosystems [61,62]. Soil microorganisms participate extensively in the decomposition and utilization of organic carbon substrates and are therefore closely associated with rhizosphere nutrient dynamics [63,64]. In the present study, variation in carbon-cycling microbial taxa and functional potential between SR and AR coincided with differences in rhizosphere nutrient conditions. These patterns suggest potential associations among microbial community composition, carbon-metabolic potential, and nutrient cycling processes within the rhizosphere [65,66].

Rhizosphere carbon metabolism has also been associated with plant metabolic status through its relationships with carbon availability and nutrient acquisition [67,68]. Microbial carbon transformation may influence the distribution and availability of carbon resources in the rhizosphere and is potentially associated with the accumulation of plant secondary metabolites, including flavonoids and phenolic compounds [69]. In the present study, differences in microbial functional potential were accompanied by substantial variation in leaf metabolite profiles between SR and AR, particularly among flavonoids, phenolic acids, and other quality-related metabolites. These coordinated patterns suggest potential links between rhizosphere microbial functional characteristics and leaf metabolic variation.

Most previous studies on rhizosphere microbial carbon metabolism have focused on fertilization, soil amendments, or plant genotype [70,71]. In contrast, the present study showed that tea plants with different propagation histories also exhibited differences in rhizosphere microbial functional and carbon-cycling profiles under comparable long-term management conditions. Overall, the observed correlations revealed associations among rhizosphere microbial composition, carbon-cycling potential, and leaf metabolic variation. Further analyses incorporating specific functional marker genes, such as *nifH*, *phoD*, and *cbh*, may help improve the functional resolution of microbial responses associated with different propagation histories.

### 4.4. Soil–Microbiome–Metabolome Interactions

The present study revealed coordinated differences in soil properties, rhizosphere microbial communities, microbial functional profiles, and leaf metabolite accumulation between SR and AR tea plants. Compared with AR, SR was associated with higher levels of several rhizosphere nutrients, distinct microbial community and functional profiles, and differences in the accumulation of quality-related metabolites. These coordinated patterns suggest associations among propagation history, rhizosphere ecological characteristics, and tea quality-related traits.

Increasing evidence indicates that plant performance is closely associated with interactions among soil conditions, microbial communities, and plant metabolic processes [72,73]. Soil nutrients provide essential resources for plant growth and metabolism, while rhizosphere microorganisms participate in nutrient transformation and resource turnover, and plant metabolic processes determine the synthesis and accumulation of quality-related compounds [74]. In the present study, higher SOM, TN, AN, and AP levels in SR coincided with differences in microbial taxa involved in organic matter turnover and nutrient cycling. The greater relative abundances of Actinomycetota and Pseudomonadota in SR were also accompanied by variation in microbial carbon-cycling potential and leaf metabolite accumulation. Together, these observations indicate coordinated associations among rhizosphere nutrient conditions, microbial community composition, microbial functional potential, and leaf metabolic profiles.

In the present study, SR was associated with higher SOM, TN, AN, and AP levels, together with differences in microbial taxa involved in organic matter turnover and nutrient cycling. The enrichment of Actinomycetota and Pseudomonadota coincided with variation in microbial carbon-cycling potential and significant differences in metabolite accumulation patterns, indicating coordinated associations among rhizosphere nutrient conditions, microbial community composition, microbial functional potential, and leaf metabolite accumulation. Correlation and co-occurrence network analyses further identified associations among dominant microbial taxa, carbon-cycling pathways, and quality-related metabolites, providing additional evidence of coordinated variation between rhizosphere microbial characteristics and leaf metabolic profiles. Differentially accumulated metabolites were identified using the criteria of VIP ≥ 1 and |log_2_FC| ≥ 1, and their biological interpretation was based primarily on coordinated metabolite-class patterns, KEGG pathway enrichment, and integrated microbiome–metabolome associations rather than on individual metabolites alone. Similar associations among soil properties, microbial communities, and plant metabolomes have also been reported in several crop systems [75,76], further supporting an integrated soil–microbiome–metabolome perspective on propagation-associated variation in tea quality.

Taken together, the observed associations among rhizosphere nutrient conditions, microbial community composition, microbial functional potential, and leaf metabolic profiles provide an integrated soil–microbiome–metabolome perspective on differences between SR and AR tea plants. The present study was conducted at a single experimental station under comparable long-term field management conditions with independent biological replicates. Future studies incorporating additional experimental sites, larger sample sizes, and controlled validation experiments would help further evaluate the generality and ecological basis of these associations.

## 5. Conclusions

This study revealed that tea plants with different propagation histories exhibited differences in soil properties, rhizosphere microbial characteristics, and leaf metabolic profiles. Compared with AR, SR showed higher levels of soil organic matter, nitrogen, and phosphorus availability, together with greater accumulation of quality-related metabolites, particularly tea polyphenols and soluble sugars. SR also exhibited higher antioxidant enzyme activities and distinct rhizosphere microbial community characteristics, including variations in taxa associated with nutrient cycling and microbial carbon metabolism. Integrated microbiome–metabolome analyses further revealed associations among SR-associated microbial taxa, carbon-metabolic pathways, and quality-related metabolites. These findings suggest that differences between SR and AR tea plants are associated with variation in rhizosphere nutrient conditions, microbial functional profiles, and quality-related compound accumulation, providing an integrated soil–microbiome–metabolome perspective on propagation-associated variation in tea quality. Further studies using controlled experiments and functional validation are needed to elucidate the mechanisms underlying these associations.

## Data Availability

The metagenomic sequencing data generated in this study are publicly available in the NCBI Sequence Read Archive (SRA) under BioProject accession number PRJNA1499368. The metabolomic data generated in this study are publicly available in the MetaboLights repository under accession number MTBLS15257.
